# Saracatinib Fails to Reduce Alcohol-Seeking and Consumption in Mice and Human Participants

**DOI:** 10.3389/fpsyt.2021.709559

**Published:** 2021-08-31

**Authors:** Summer L. Thompson, Carol A. Gianessi, Stephanie S. O'Malley, Dana A. Cavallo, Julia M. Shi, Jeanette M. Tetrault, Kelly S. DeMartini, Ralitza Gueorguieva, Brian Pittman, John H. Krystal, Jane R. Taylor, Suchitra Krishnan-Sarin

**Affiliations:** ^1^Department of Psychiatry, Yale University School of Medicine, New Haven, CT, United States; ^2^Interdepartmental Neuroscience Program, Yale University Graduate School of Arts and Sciences, New Haven, CT, United States; ^3^Program in Addiction Medicine, Department of Internal Medicine, Yale University School of Medicine, New Haven, CT, United States; ^4^Department of Biostatistics, Yale University School of Public Health, New Haven, CT, United States; ^5^Department of Neuroscience, Yale University School of Medicine, New Haven, CT, United States; ^6^Department of Psychology, Yale University, New Haven, CT, United States

**Keywords:** saracatinib, AZD0530, Fyn kinase, alcohol use disorders, alcohol habit, NMDA receptor, glutamate, AM404

## Abstract

More effective treatments to reduce pathological alcohol drinking are needed. The glutamatergic system and the NMDA receptor (NMDAR), in particular, are implicated in behavioral and molecular consequences of chronic alcohol use, making the NMDAR a promising target for novel pharmacotherapeutics. Ethanol exposure upregulates Fyn, a protein tyrosine kinase that indirectly modulates NMDAR signaling by phosphorylating the NR2B subunit. The Src/Fyn kinase inhibitor saracatinib (AZD0530) reduces ethanol self-administration and enhances extinction of goal-directed ethanol-seeking in mice. However, less is known regarding how saracatinib affects habitual ethanol-seeking. Moreover, no prior studies have assessed the effects of Src/Fyn kinase inhibitors on alcohol-seeking or consumption in human participants. Here, we tested the effects of saracatinib on alcohol consumption and craving/seeking in two species, including the first trial of an Src/Fyn kinase inhibitor to reduce drinking in humans. Eighteen male C57BL/6NCrl mice underwent operant conditioning on a variable interval schedule to induce habitual responding for 10% ethanol/0.1% saccharin. Next, mice received 5 mg/kg saracatinib or vehicle 2 h or 30 min prior to contingency degradation to measure habitual responding. In the human study, 50 non-treatment seeking human participants who drank heavily and met DSM-IV criteria for alcohol abuse or dependence were randomized to receive 125 mg/day saracatinib (*n* = 33) or placebo (*n* = 17). Alcohol Drinking Paradigms (ADP) were completed in a controlled research setting: before and after 7–8 days of treatment. Each ADP involved consumption of a priming drink of alcohol (0.03 mg%) followed by *ad libitum* access (3 h) to 12 additional drinks (0.015 g%); the number of drinks consumed and craving (Alcohol Urge Questionnaire) were recorded. In mice, saracatinib did not affect habitual ethanol seeking or consumption at either time point. In human participants, no significant effects of saracatinib on alcohol craving or consumption were identified. These results in mice and humans suggest that Fyn kinase inhibition using saracatinib, at the doses tested here, may not reduce alcohol consumption or craving/seeking among those habitually consuming alcohol, in contrast to reports of positive effects of saracatinib in individuals that seek ethanol in a goal-directed manner. Nevertheless, future studies should confirm these negative findings using additional doses and schedules of saracatinib administration.

## Introduction

Alcohol is a leading public health problem, presenting the largest risk factor for premature death for young to middle aged adults worldwide (1). Alcohol use disorder (AUD) is the most prevalent substance use disorder other than tobacco use disorder, yet currently available treatments are rarely used (1, 2). Three pharmacotherapies for AUD have U.S. Food and Drug Administration approval: disulfiram, naltrexone (oral and long-acting injectable), and acamprosate (2). However, these agents have issues of modest efficacy, adherence, and possible restricted effect to subpopulations (3, 4), which highlights the need for novel AUD treatment options.

The glutamatergic system is heavily implicated in the pathophysiology of AUD, providing potential targets for novel therapeutics (5, 6). Indeed, pharmacological manipulation of AMPA, kainate, mGlu, and NMDA glutamate receptors (NMDAR) can alter alcohol consumption, seeking, withdrawal or reinstatement (5, 7–14). The NMDAR is one of the highest affinity targets of ethanol in the brain (15), and chronic ethanol exposure is associated with altered NMDAR signaling (16–18). NMDARs play a role in various consequences of chronic alcohol use (19): NMDAR antagonists can reduce ethanol tolerance, craving/seeking, and consumption (20–24). For example, the uncompetitive NMDAR antagonist memantine reduces cue- and alcohol-induced craving in humans (7, 25) and we have also observed that a low dose of memantine combined with a standard dose of the opioid antagonist naltrexone was well-tolerated and resulted in reduced alcohol drinking and craving within a sample of individuals with a positive family history of AUD (21). Our earlier work has also observed that only lower doses of memantine reduce alcohol craving, whereas higher doses increase alcohol consumption, especially in individuals with high levels of baseline impulsivity (26). NMDAR antagonists can have undesirable cognitive and psychotomimetic effects (27, 28). Together, this evidence suggests that NMDARs may be a promising target for amelioration of the hyper-glutamatergic state in AUD, but that direct antagonism may present challenges and more nuanced approaches that target this system may be needed (5, 21).

Fyn is an Src family protein tyrosine kinase that indirectly upregulates NMDAR activity by phosphorylating the NR2B subunit, a component of the NMDAR that is particularly implicated in the molecular and behavioral adaptations to chronic ethanol exposure (29–31). Mounting evidence implicates Fyn in alcohol use behaviors in human participants and rodents. Multiple studies have identified polymorphisms in the Fyn gene associated with increased risk for AUD (32–34). Rodent studies revealed that ethanol activates Fyn in the dorsomedial striatum (DMS) (35–38). The DMS is a key brain region for goal-directed action, which refers to behaviors that are sensitive to changes in action-outcome contingencies (39). Furthermore, ethanol-induced long-term facilitation in the DMS is Fyn-dependent (36, 37). Importantly, pharmacological inhibition of Fyn using the Src/Fyn kinase inhibitor saracatinib (AZD0530) was reported to reduce ethanol-seeking and enhance extinction of ethanol-seeking in mice with goal-directed responding for ethanol (35) and reduce ethanol consumption in ethanol-naïve mice (40), suggesting that saracatinib may be a viable treatment option for goal-directed drinking.

Habits, in contrast to goal-directed behaviors, are insensitive to changes in action-outcome contingencies or devaluation of previously desirable outcomes and reflect a shift from recruitment of DMS to dorsolateral striatum (DLS) (39, 41–43). Ethanol cues can disrupt otherwise goal-directed food-seeking, and chronic ethanol exposure facilitates the development of food habits (41, 44). Ethanol-seeking transitions from goal-directed to habitual more readily than food-seeking (45–48). Indeed, overreliance on habits is thought to contribute to compulsive drug-seeking including in AUD (45, 49, 50), and has been observed in individuals with AUD (51). However, no studies have examined the efficacy of saracatinib for reducing ethanol-seeking and consumption in habitual alcohol consumers. Here, we performed two parallel studies in mice and human participants to assess the ability of saracatinib to reduce alcohol consumption and seeking/craving in habitual ethanol-seeking mice and participants who were heavy drinkers with an AUD.

## Materials and Methods

### Mouse Study

#### Mice

Eighteen adult male C57BL/6NCrl mice (Charles River Laboratories, Wilmington, MA) were used for the mouse experiment. Mice were delivered at 8–9 weeks old and allowed to acclimate to the vivarium for 7 days before initiating food restriction to 85–90% of free-feeding body weight. Mice had *ad libitum* access to water in the home cage but were provided with their daily food 15 min prior to initiating operant sessions without water access to induce thirst. Mice were pre-exposed to 10% ethanol, 0.1% saccharin solution in the home cage for 1 h, 2 days in a row prior to initiating operant training with 10% ethanol, 0.1% saccharin as the reinforcer (10 μl per reward). All procedures were approved by the Yale University Institutional Animal Care and Use Committee and in accordance with the National Institutes of Health *Guide for the Care and Use of Laboratory Animals of the Institute of Animal Resources*.

#### Mouse Drugs

Saracatinib, also known as AZD0530, was obtained from AstraZeneca, Boston, MA. Saracatinib was dissolved in saline and administered at a dose of 5 mg/kg. This dose was based on preliminary studies showing that this dose reduces NR2B phosphorylation in the DMS (data not shown) and to match levels of saracatinib in cerebrospinal fluid with that expected for the human study (52), which was performed simultaneously. AM404 (R&D Systems, Minneapolis, MN) is an endocannabinoid transport inhibitor that we have previously shown to reduce habitual responding for ethanol (53): it was used as a positive control for testing the malleability of habitual ethanol-seeking. AM404 was dissolved in 5% DMSO, 15% Tween 80 in sterile physiological saline and administered at a dose of 10 mg/kg body weight. Drugs were administered via intraperitoneal injection (i.p.) at 10 ml/kg body weight.

#### Mouse Behavioral Paradigm

##### Apparatus and Training

Mice were trained and tested in standard mouse operant conditioning chambers in sound attenuation cabinets (Med Associates, St. Albans, VT). Chambers were equipped with three nose port apertures and a magazine with photobeam sensors to record entries and lights to indicate active ports. Ethanol reinforcers (10% ethanol v/v, 0.1% saccharin) were delivered into the magazine using a dipper arm holding a 10 μl cup that was submerged in a reservoir of the reinforcer solution and would then raise the cup through a hole into the magazine to deliver the reinforcer, which was provided for 10s before retraction of the arm back into the reservoir. Mice were trained daily in the same operant chamber throughout the experiment.

Mice first learned to associate the magazine with reinforcer delivery in two 40-min magazine training sessions. Each session began with a reinforcer delivered into the magazine 60 s into the session. This reinforcer remained available (i.e., dipper arm raised with cup accessible inside the magazine) until the mouse entered the magazine, and then for the subsequent 10 s before the dipper arm was retracted. Following this non-contingent delivery, reinforcers were delivered on a fixed interval-60 s schedule throughout the session, meaning that following a minimum of 60 s, the next magazine entry elicited a reinforcer delivery.

Next mice were trained to perform the operant response on a fixed ratio-1 (FR-1) schedule. One nose port was designated the “active” port for that mouse (left or right), counterbalanced between animals but consistent between sessions. The active port was indicated by illumination of the port. Sessions began with a single non-contingent reinforcer. Just like magazine training, reinforcers remained available until the animal entered the magazine, after which the dipper was available for 10 s before retraction. Following this free reinforcer, entries into the active nose port resulted in delivery of a single reinforcer. FR-1 sessions lasted 45 min or until the mouse earned 60 reinforcers, whichever occurred first. Mice completed FR-1 training upon reaching a criterion of 13 reinforcers within a single session.

Following FR-1 training, mice earned ethanol reinforcers on a variable interval (VI) schedule that we have previously shown to promote habitual responding for ethanol (53). The same active nose port assigned during FR-1 training remained the active port for each mouse during VI sessions, as indicated by illumination of the active port throughout the session. Intervals were selected pseudo-randomly from an exponential array that averaged to the schedule duration, after which the first active nose port response resulted in a reinforcer, as previously (53). Unlike during magazine and FR-1 training, these reinforcers remained available for the subsequent 10 s following the active nose port response, regardless of whether the mouse had yet entered the magazine. Sessions lasted 45 min. Mice were trained on a VI-30 schedule for 3 days, followed by VI-60 for ~24 days.

##### Contingency Degradation

Contingency degradation sessions delivered ethanol reinforcers non-contingently at the same rate that mice earned rewards in the previous VI session. Active nose port entries had no programmed responses. Reinforcer delivery occurred at equal intervals that were individually tailored to the prior day reinforcement rate of that mouse, meaning each mouse received the same number of reinforcers as in the prior day VI session. Sessions lasted 45 min. Mice underwent multiple contingency degradation sessions to test effects of pharmacological agents. Initial testing occurred following a minimum of 20–25 days of VI-60 training. Between contingency degradation tests, mice underwent additional VI-60 training days to stabilize responding. Response rates, magazine entries, and incentivized entries (i.e., magazine entries while reinforcer is available) were measured and compared between the contingency degradation test and the preceding day's VI-60 session, which was used as a baseline. The amount of ethanol consumed relative to body weight was estimated based on the number of reinforcers earned. However, consumption could not be directly confirmed due to the design of the reinforcer delivery apparatus, which resubmerged the dipper cup into the reservoir after each reinforcer to refill the cup for the subsequent reinforcer.

##### Pharmacological Testing

For each contingency degradation test, the vehicle solution for the pharmacological agent was administered prior to the baseline VI-60 session. The day after completing the baseline session, mice received pharmacological challenge and underwent contingency degradation testing. First, all animals (*n* = 18) received AM404 or vehicle 30 min prior to the contingency degradation test session in a within-subject, counterbalanced manner. This test served to: (1) provide confirmation that the group exhibited habitual responding for ethanol (i.e., lack of decrease in responses during contingency degradation under vehicle conditions) and (2) provide a positive control testing whether the habitual responding was sensitive to goal-directed-promoting agents, as we have previously shown that AM404 reduces habitual responding for ethanol (53). AM404 was tested within-subject based on our previous experience with this drug not showing cross-over effects (53, 54). Following stabilization of responding on the VI-60 schedule following these contingency degradation tests, saracatinib was tested in a between-subject cross-over design, in which half the animals received saracatinib for the 2-h pretreatment condition (*n* = 8/drug), which occurred first for all animals, whereas the other half received saracatinib for the 30-min pretreatment condition (*n* = 8/drug), which occurred second for all animals. One animal was excluded in each drug group in each time point due to computer error for a final *n* = 8/group. The 2-h pretreatment schedule was selected based on our preliminary studies showing reduced free-access ethanol consumption in the home cage at this time point (data not shown) and the 30-min pretreatment schedule was designed to match the effective time point for AM404 (53). Overall, animals received one administration of AM404 vehicle and AM404 prior to any saracatinib administration, and then all mice received one dose of saracatinib, at either a 2-h or 30-min pretreatment time point.

#### Statistical Analyses

Data were analyzed using SPSS 26 (IBM, Armonk, NY) and graphed using Prism 8 (Graphpad, San Diego, CA). Outcome measures included active responses, total magazine entries, and incentivized entries, which were assessed using generalized estimating equations with a Poisson distribution with Wald's chi square test statistics. Significant interactions were resolved by making pairwise comparisons of the estimated marginal means corrected for multiple comparisons using Sidak's method. Alpha was set to a threshold of 0.05.

### Human Clinical Trial

#### Human Participants

Participants (*n* = 50 randomized to treatment; *n* = 33 saracatinib, *n* = 17 placebo) were non-treatment seeking, heavy drinkers that met the DSM-IV criteria for alcohol abuse or dependence (Table 1; Supplementary Figure 1). Additional inclusion criteria were: between 21 and 50 years of age, body mass index between 19 and 30, capable of reading English at the 6th grade level or above, average weekly alcohol consumption of 25–70 standard drinks for men and 20–65 for women with no more than 3 days of abstinence per week during the month prior to the intake [Timeline Follow-Back method; TFLB; (55)]. Exclusion criteria included medical contraindications to drinking alcohol or use of saracatinib, abuse or dependence on substances other than alcohol or nicotine, severe psychiatric disability, significant alcohol withdrawal at any intake appointment [Clinical Institute Withdrawal Assessment for Alcohol Scale score > 8 (56)], current use of psychoactive drugs or CYP3A4 inhibitors or warfarin, those who were not on stable use of prescribed antidepressants/anxiolytics, those who reported disliking spirits or were seeking treatment for their drinking, and those who were pregnant or nursing.

**Table 1:** Participant demographics and drinking histories.

	**All Participants (*n* = 50)**	**Placebo (*n* = 17)**	**Saracatinib (*n* = 33)**	***P***
**Demographics**				
Male, *n* (%)	25 (50%)	9 (53%)	16 (48%)	0.77
Current smokers, *n* (%)	19 (39%)	7 (44%)	12 (36%)	0.62
White, *n* (%)	31 (62%)	10 (59%)	21 (64%)	0.74
Family Hx positive, *n* (%)	20 (40%)	7 (41%)	13 (39%)	0.90
Age, mean (SD)	29 (7.8)	30 (7.9)	29 (7.8)	0.49
**Drinking based on 30-day timeline followback interview**				
Total # drinks, mean (SD)	171 (68)	175 (62)	169 (73)	0.75
Drinks/drinking day, mean (SD)	7.8 (2.8)	7.3 (1.8)	8.1 (3.2)	0.36
% drinking days, mean (SD)	74 (17)	79 (17)	71 (17)	0.10
Alcohol dependence score	10.7 (5.3)	9.9 (5.3)	11.2 (5.4)	0.43

*N = 50 total; n = 17 placebo and n = 33 saracatinib. There were no differences between the groups for demographics or drinking measured in the Timeline Followback interview. Hx, history; SD, standard deviation*.

#### Study Medications

Participants were randomized on a 2:1 ratio (active vs. placebo) to receive saracatinib (125 mg/day, oral) or matching placebo for seven to 8 days to achieve steady state drug levels following exposure to 4–5 half-lives of the drug (*t*_1/2_ = 40 h). The Yale New Haven Investigational Pharmacy randomized the participants and dispensed the study medications; all research staff and the participants were blind to treatment assignment. The dose was selected based on previous studies demonstrating safety and tolerability of 125 mg/day saracatinib in human participants (57) and evidence that this dose reached comparable levels in cerebrospinal fluid to that of 5 mg/kg in mice, a dose that has been shown to produce neural changes (52).

#### Study Design

This study was a randomized, double-blind, placebo-controlled trial that was approved by the Yale Human Investigations Committee, registered in ClinicalTrials.gov (NCT02955186), and followed the National Advisory Council for Alcohol Abuse and Alcoholism guidelines (58). Alcohol drinking behaviors were assessed using an established alcohol drinking paradigm (ADP) conducted in a private room at the Hospital Research Unit (HRU) of Yale New Haven Hospital (YNHH). The ADP involved consumption of a priming drinking of alcohol followed by choice *ad libitum* consumption of up to 12 drinks over three 1-h self-administration periods, as done previously (21). Participants completed a baseline ADP and were then randomized to receive saracatinib (125 mg/day) or placebo for a 7–8 day period (Supplementary Figure 2); participants were contacted daily either in person or virtually to observe medication administration and check for adverse events. At the end of this period, they completed the second, on-treatment ADP.

The YNHH Investigational Pharmacy calculated and delivered alcohol doses of each participant's preferred alcohol to the HRU; the doses were designed to raise blood alcohol levels to 0.03 g/dl for priming drink and 0.015 g/dl for all other drinks based on a formula that takes into account the sex, weight, and age of the participant (59). Each alcohol dose was mixed with the participant's preferred non-caffeinated, non-carbonated mixer in a 1:3 ratio. Each participant's preferred alcohol and mixer were determined at an earlier appointment.

Following completion of each ADP, participants spent the night at the HRU and were discharged the next morning. They also received a 1-week follow-up appointment to assess for adverse events and drinking, and a motivational intervention to discuss their alcohol use and encourage readiness to change. Participants were paid to participate and could earn up to $1,142 for completing all portions of the study.

#### Measures

##### Alcohol Craving

Craving was measured 30 min prior to the priming dose (baseline), and then 10, 20, 30, 40, and 50 min during the priming dose period and every half hour during each *ad libitum* period (i.e., 90, 120, 150, 180, 220, and 240 min) using the 8-item Alcohol Urge Questionnaire (AUQ) (60). Separate area under the curve (AUC) estimates for each phase were calculated using the trapezoidal rule based on the time points specified above.

##### Standard Drinks Consumed

Total number of standard drinks consumed during the 3-h self-administration period.

##### Alcohol-Induced Stimulation/Sedation

Determined at 10, 20, and 50 min during the priming dose period and then every hour at the end of each of the three *ad libitum* periods with the brief Biphasic Alcohol Effects Scale [BAES; (61)].

##### Adverse Events

Measured daily during the study medication period using the SAFTEE (62).

#### Statistical Analyses

Baseline demographics and drinking characteristics were compared among medication conditions using *t*-tests and chi-square tests as appropriate. Data were checked for normality and transformations applied as necessary. The two primary outcomes of interest were: craving (AUQ) and total drinks consumed during the *ad libitum* periods, each tested on an intent-to-treat (ITT) basis at the α = 0.05 threshold. Subjective craving (AUQ) was quantified by calculating an area under the curve (AUC) for each phase (priming dose, *ad libitum*) within each ADP using the trapezoidal rule, and analyzed using linear mixed models with medication (placebo, saracatinib) included as a between-subjects factor and session (baseline, on-Tx) included as a within-subjects factor. The medication by time interaction was modeled and participant was the clustering factor. Total drinks consumed was analyzed using an identical linear mixed model as described for craving. Potential confounding factors (sex, family history, age, and baseline drinking variables) were tested by including them in each model but were not significant and dropped for parsimony. Similar models were used to assess BAES outcomes. For all models, the best-fitting variance-covariance structure was based on the Schwarz-Bayesian Criterion (BIC) (63). Least-square means were estimated and plotted to determine the nature of significant effects. All analyses were performed using SAS, version 9.4 (Cary, NC).

## Results

### No Effect of 5 mg/kg Saracatinib on Habitual Responding for Ethanol in Mice

By the end of VI training, mice earned 1.04 ± 0.03 (standard error of the mean) g/kg ethanol within the final session. Although consumption could not be directly confirmed due to the refilling of the dipper cup for each reinforcer delivery, all mice entered the magazine while the dipper cup was available (i.e., incentivized entries) at least as many times as reinforcers earned, and the number of incentivized entries was significantly greater than the number of reinforcers earned [$\chi_{\left(1\right)}^{2}$ = 7.15, *p* < 0.01], suggesting knowledge of the action-outcome contingency and the opportunity to consume the ethanol reinforcers.

Following training, AM404 was administered during contingency degradation to evaluate whether animals exhibited habitual responding for ethanol, and whether responding and ethanol consumption were sensitive to drug challenge with a known enhancer of goal-directed response patterns. As expected, AM404 reduced the number of active responses during the contingency degradation whereas vehicle administration did not affect responding (Supplementary Figure 3). These results suggest that the mice were sufficiently trained to respond habitually for ethanol, and that AM404 successfully reduced habitual responding for ethanol, consistent with our previous work (53).

**Figure 1:**
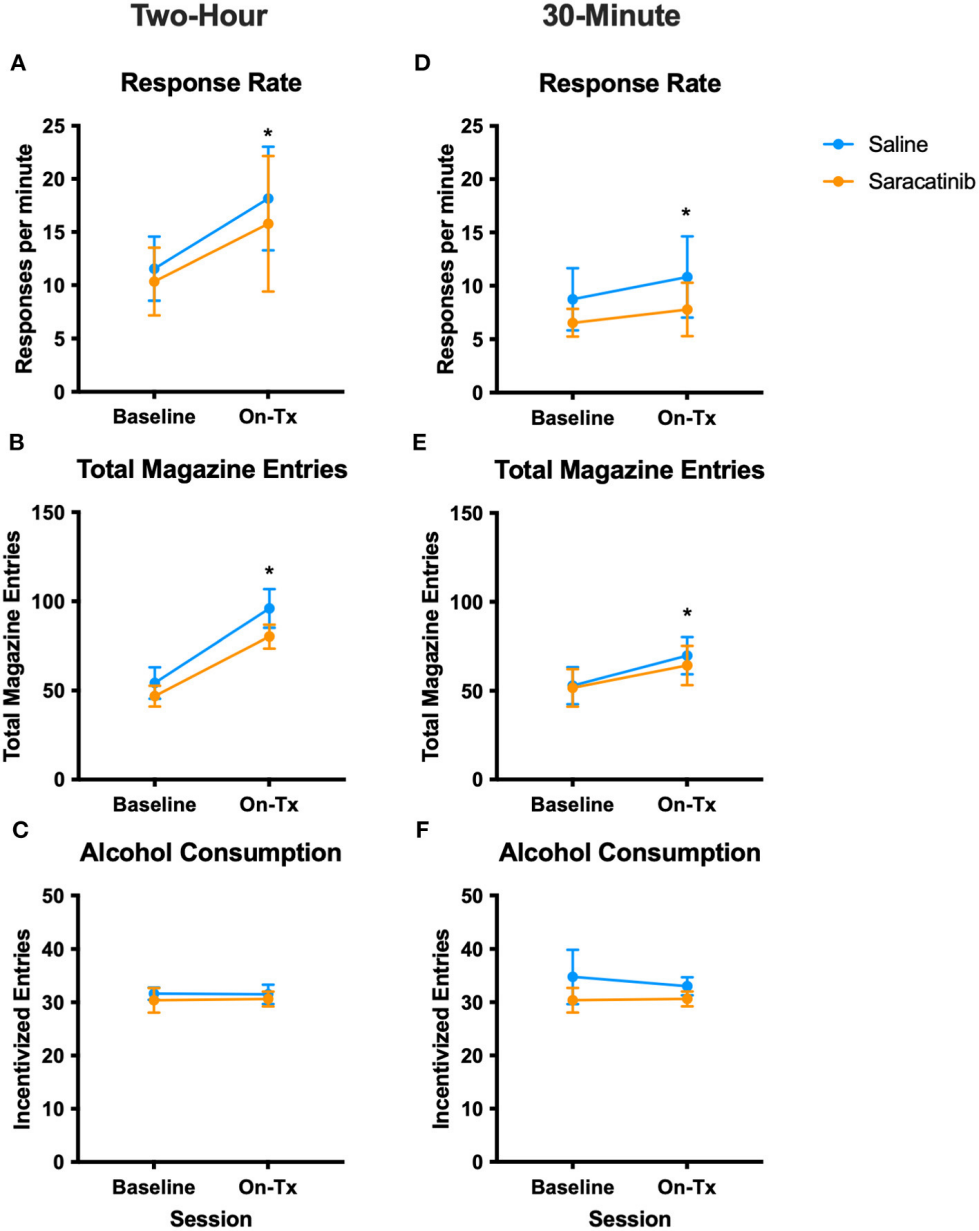
5 mg/kg saracatinib did not affect habitual ethanol-seeking or consumption in mice at either time point. Mice received an i.p. injection of saline 2 h **(A–C)** or 30 min **(D–F)** prior to the VI-60 session preceding contingency degradation (“Baseline”). The following day, mice received an i.p. injection of saline (control condition) or saracatinib 2 h **(A–C)** or 30 min **(D–F)** prior to the contingency degradation session (“On-Tx”). **(A–C)** Response rate, total magazine entries, and incentivized magazine entries for the 2-h time point, respectively. **(D–F)** Response rate, total magazine entries, and incentivized magazine entries for the 30-min time point. Two-hour time point: *n* = 8/drug. Thirty-minute time point: *n* = 8/drug. *p < 0.05 vs. baseline day across groups (main effect of session). Tx, treatment; i.p., intraperitoneal.

Next, we sought to determine whether saracatinib could also reduce habitual responding for ethanol. A dose of 5 mg/kg saracatinib was administered 2 h prior to the contingency degradation test and did not significantly reduce habitual responding for ethanol (Figure 1A). An increase in responding was observed across groups during contingency degradation relative to baseline [$\chi_{\left(1\right)}^{2}$ = 37.01, *p* < 0.0001]. Likewise, magazine entries were increased across groups during contingency degradation [$\chi_{\left(1\right)}^{2}$ = 46.22, *p* < 0.0001; Figure 1B]. Finally, no effects of session or drug were identified for incentivized magazine entries (Figure 1C), a measure of ethanol-seeking behavior (53). Consistent with the amount of ethanol delivered during VI training, mice received an average of 1.17 ± 0.03 (standard error of the mean) g/kg ethanol during testing, wherein an identical number of reinforcers were delivered during the baseline VI-60 and contingency degradation sessions. Overall, saracatinib did not alter habitual responding for ethanol or ethanol consumption when administered 2 h prior to contingency degradation testing.

Next, we sought to determine whether the lack of effect of saracatinib identified at the 2-h time point was due to a suboptimal time point. We assessed whether a 30-min pretreatment time point, the time point used for the positive control compound AM404, would reveal effects of saracatinib on habitual ethanol responding. Consistent with the 2-h pretreatment, mice increased active responding during contingency degradation across drug groups [$\chi_{\left(1\right)}^{2}$ = 4.45, *p* < 0.05], but no effects of saracatinib were identified (Figure 1D). Magazine entries increased during contingency degradation across drug groups [$\chi_{\left(1\right)}^{2}$ = 6.33, *p* < 0.05; Figure 1E]. No effects of saracatinib or session type were identified for incentivized magazine entries (Figure 1F). Consistent with prior testing phases, mice received an average of 1.11 ± 0.03 (standard error of the mean) g/kg ethanol. Overall, saracatinib did not affect habitual responding for ethanol when administered 30 min prior to contingency degradation testing.

### No Effect of 125 mg/day Saracatinib on Alcohol Craving, Alcohol-Induced Stimulation/Sedation, or Alcohol Consumption in Human Participants

The final sample of randomized participants (Table 1) included 25 men and 25 women, with an average age of 29.0 [standard deviation (SD) = 7.8], a diverse racial distribution (31 White, 17 Black, 2 other), and 19 individuals who currently smoked tobacco (39%), with mean scores of 12.1 (SD = 5.6) on the Alcohol Dependence Scale (64). During the 30 days prior to the baseline ADP, participants consumed, on average 171 (SD = 68) drinks, 7.8 drinks per drinking occasion (SD = 2.8) and drank 3 out of every 4 days (74%, SD = 17%). No differences in demographic variables were observed between the saracatinib and placebo groups. See Supplementary Figure 2 for CONSORT diagram.

Saracatinib was well-tolerated and we did not observe any serious adverse events. The most common adverse events reported included nausea (saracatinib: *n* = 5, 15%; placebo: *n* = 1, 6%) and headache (saracatinib: *n* = 5, 15%; placebo: *n* = 1, 6%). As shown in Supplementary Table 1, participants who received saracatinib also reported other gastrointestinal symptoms such as abdominal discomfort and diarrhea (*n* = 3, 9%), as well as cold symptoms (*n* = 6, 18%), nasal congestion (*n* = 4, 12%) and joint pain (*n* = 3, 9%). No one dropped out of the study due to adverse events. For detailed information on adverse events see Supplementary Tables 1, 2.

**Figure 2:**
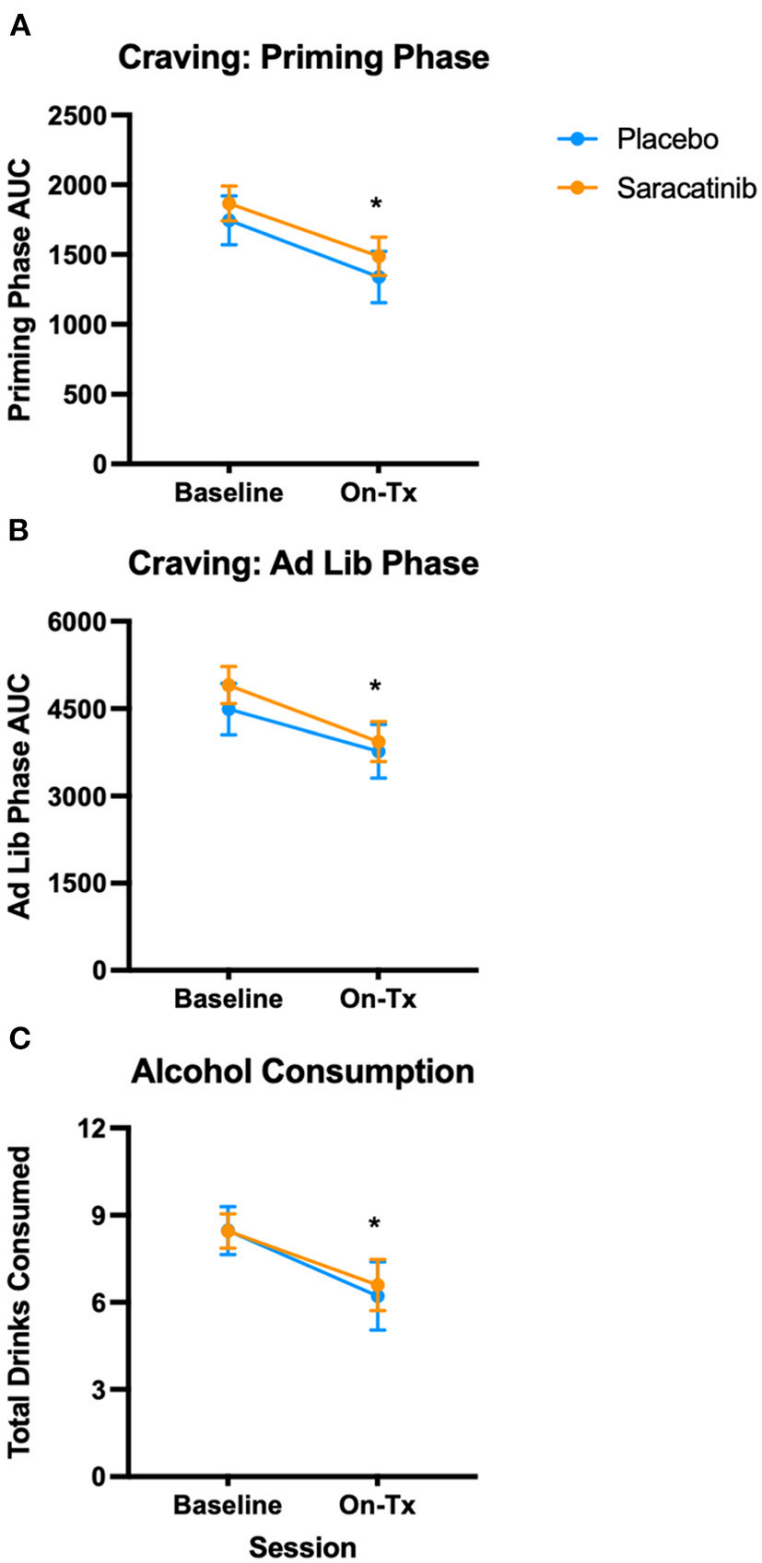
125 mg/day saracatinib did not affect alcohol craving or consumption in human participants. Participants underwent two ADPs: One prior to initiating treatment (“Baseline”) and a second one following 7–8 days of 125 mg/day oral saracatinib treatment (“On-Tx”). **(A)** AUC for craving during the priming phase of the ADP session. **(B)** AUC for craving during the *ad libitum* phase. **(C)** Total drinks consumed. *N* = 15 placebo; *n* = 26 saracatinib. *p < 0.05 vs. baseline ADP across groups (main effect of session). ADP, alcohol drinking paradigm; AUC, area under the curve; Tx, treatment.

Estimated least-square means and standard errors depicting the effects of saracatinib on craving for alcohol are shown in Figures 2A,B. Reductions in craving from baseline were observed across the placebo and saracatinib treatments during both the priming dose phase [Figure 2A; F_(1,39)_ = 11.8, *p* = 0.0014] and the *ad libitum* drinking phase [Figure 2B; F_(1,39)_ = 10.1, *p* = 0.003]. However, the observed patterns of reductions in craving were similar among medications during the priming dose [F_(1,39)_ = 0.01, *p* = 0.91] and *ad libitum* drinking [F_(1,39)_ = 0.21, *p* = 0.65] phases of the paradigm. Craving was not associated with any of the considered baseline covariates.

Similar to measures of craving, total drinks consumed (Figure 2C) showed an overall 25% reduction from the baseline ADP (8.5 ± 0.51 (standard error of the mean) to the on-treatment ADP session (6.4 ± 0.73) [F_(1,39)_ = 10.9, *p* = 0.002], but the reductions did not differ by medication [medication by session: F_(1,39)_ = 0.10, *p* = 0.75].

We did not observe significant effects of saracatinib on alcohol-induced stimulation or sedation measured using the BAES (data not shown).

## Discussion

In the present animal and human studies, we assessed the possibility of a role for Fyn in habitual alcohol-seeking and drinking in both mice and humans using the Fyn kinase inhibitor saracatinib. Overall, we did not identify effects of saracatinib in either mice or humans, suggesting that saracatinib, at the doses tested, may not be an effective treatment for reducing alcohol-seeking or consumption in individuals who habitually consume alcohol.

In mice, we used our established, extended instrumental training paradigm to induce habitual responding for ethanol and assessed the effects of acute administration of saracatinib on ethanol habit. We first demonstrated that this habitual responding for ethanol was sensitive to pharmacological manipulation by administering a positive control compound, AM404, an endocannabinoid transport inhibitor that we have previously shown to reduce habitual responding for ethanol (53). AM404 successfully reduced habitual ethanol-seeking, indicating that the habitual ethanol-seeking was receptive to pharmacological manipulation. However, 5 mg/kg saracatinib failed to alter habitual responding for ethanol in mice. This lack of effect was not likely to be due to time of saracatinib administration, as neither 2-h, nor 30-min pretreatment was sufficient to alter habitual ethanol-seeking in these mice. These time points encompass the 1-h pretreatment employed in a study that showed saracatinib-induced reduction in ethanol self-administration in mice reported to have goal-directed responding for ethanol (35). Furthermore, saracatinib is long-lasting in the mouse brain, with a half-life of approximately 16 h (52). Moreover, Fyn activity is upregulated in as little as 15 min (37) and for as long as 16 h (36) following ethanol exposure in rodents. Overall, these findings suggest that acute administration of 5 mg/kg saracatinib does not modulate ethanol habit in mice.

In the human clinical trial, we assessed alcohol craving and consumption in non-treatment seeking participants with heavy drinking habits using our established ADP paradigm before and after saracatinib administration. No effects of 7–8 days of oral 125 mg saracatinib were identified for craving in either the priming or *ad libitum* consumption phases. Furthermore, no effects of saracatinib were observed for the number of drinks consumed. Of note, we observed a reduction in drinking and craving in the placebo group and in the saracatinib group. While it is possible that the decrease in the placebo group could have masked any effects of saracatinib, we have demonstrated drug-placebo differences in other studies using this ADP paradigm (21). Together, these results suggest that short-term saracatinib treatment at a dose of 125 mg/day may not reduce alcohol craving or consumption in people with heavy drinking habits.

The doses used in these studies were selected based on several factors: to match cerebrospinal fluid levels of saracatinib between the two species (52), verified behavioral effects and peripheral markers of reduced Src family activity (52, 57), and to mitigate risk of off-target pharmacological effects and side effects (57, 65). It is possible that alternative doses of saracatinib would yield different results in both species. Of note, the rate of adverse events observed, including neuropsychiatric adverse events, with the 125 mg/day saracatinib dose in human participants was low compared to what is commonly seen with glutamatergic agents (21). In contrast, larger clinical trials in older clinical populations with Alzheimer's disease (66) have identified higher rates of adverse effects within the range of the dose used in our study. So, our observed lack of adverse events and efficacy may be related to the population studied, which may potentially tolerate, and require, higher doses to reverse alcohol-induced glutamatergic changes due to heavy drinking habits. For example, in mice, studies that used a dose of 10 mg/kg have reported saracatinib-mediated reductions in ethanol self-administration (35). However, in our preliminary work (data not shown) 5 mg/kg of saracatinib was sufficient to reduce phosphorylated NR2B in DMS, and several studies have used this dose to successfully ameliorate behavioral deficits or neurodegeneration in Alzheimer's models, albeit administered per oral and on a chronic treatment regimen (52, 67, 68). Nonetheless, future studies should perform a dose-response curve for effects of saracatinib on habitual responding for alcohol in mice to elucidate the present negative findings. Overall, further work to examine the dose-dependent effects of saracatinib on alcohol drinking behaviors is needed.

Another possibility for the lack of treatment effects is the time course of the treatment regimen. While positive effects of saracatinib on ethanol-seeking and consumption have been reported after acute administration in mice (35, 40), other behavioral effects of saracatinib, at the 5 mg/kg dose used in the present study, required longer time frames. For example, rescue of cognitive function in an Alzheimer's mouse model required 3–5 weeks of 5 mg/kg saracatinib administration for effects to emerge (52). Likewise, it is possible that a more extended treatment regimen in the clinical trial would have yielded positive results. Indeed, maximal plasma levels are augmented at steady state relative to acute administration at the 125 mg/day dose, with participants reaching steady state within 10–17 days (65), whereas the present study provided saracatinib for 7–8 days. However, a clinical trial that assessed the efficacy of saracatinib in Alzheimer's disease did not observe significant effects at this dose after a year of treatment, despite positive effects within shorter timeframes in mouse models (52, 66). Regardless, it is possible that extended treatment regimens may be needed when considering the use of this agent to treat alcohol drinking, which may yield different findings.

Alternatively, Fyn may have brain region- and function-specific roles that explain the present results. Fyn-dependent long term facilitation of NMDAR-mediated excitatory postsynaptic currents in response to ethanol are observed in the DMS, but not DLS (36, 37). The same study found that the Src family protein tyrosine kinase inhibitor PP2, which inhibits Fyn, reduced ethanol self-administration in rats when infused into the DMS, but not DLS. Furthermore, it was recently reported that stimulation of D1 neurons in the DMS, but not DLS upregulated phosphorylation of Fyn and its substrate NR2B (40), together suggesting that Fyn may play less of a role in the DLS. These findings align with the possibility that Fyn may mediate goal-directed, but not habitual, ethanol-seeking and consumption behaviors; the DMS is classically implicated in goal-directed action, whereas there is a lateral shift in activity over time as an action becomes more habitual, including ethanol-seeking (39, 41, 42). This possibility is supported by the current literature regarding effects of saracatinib on ethanol-seeking and consumption. One study reported a reduction in instrumental responding for ethanol in confirmed goal-directed mice after acute administration of saracatinib (35). Another study from the same group reported reductions in *ad libitum* ethanol consumption in ethanol-naïve mice (40). However, we did not test effects of saracatinib on goal-directed drinking in the present study, and thus cannot confirm this selectivity from the present results alone. To our knowledge, the current studies are the first to directly assess the effects of saracatinib in confirmed habitual ethanol consuming individuals, who likely have greater DLS control of ethanol-seeking (41, 69).

While a strength of these parallel studies is the use of equivalent doses of saracatinib in chronically alcohol consuming individuals, there are disparities between the designs of the mouse and human studies that limit the comparability. Saracatinib was administered acutely in the mouse study, whereas the human participants received 7–8 days of saracatinib. In addition, only male subjects were used in the mouse study. Furthermore, the mice were not tested on measures of ethanol dependence, and blood ethanol concentrations were not measured, which precludes classification of these mice as heavy drinkers or as ethanol-dependent. However, prior studies have reported binge-level blood ethanol concentrations in mice consuming similar quantities of ethanol during self-administration. For example, one study reported an average blood ethanol concentration of 93 mg/dl after consuming 1.3 g/kg ethanol within a 60-min session, which may be comparable to the present study in which mice received approximately 1.1 g/kg ethanol within a 45-min session (70). In addition, in our other studies using this self-administration paradigm we have observed heightened withdrawal-induced aggression between male cage mates (data not shown), which suggests that this experimental setup may be capable of inducing ethanol dependence. Nonetheless, these features must be quantified in future studies for confirmation.

Another key difference between these studies was the direct assessment of habitual behavior in the mice, which was not tested in the clinical study. Previous studies have shown that alcohol-dependent individuals exhibit a shift toward more habitual, less goal-directed behavior in an outcome devaluation test (51). Furthermore, another study found that habitual, but not reward-driven alcohol use was associated with severity of alcohol dependence (71), and another found that abstinent participants with high alcohol expectancies and impaired goal-directed control were more susceptible to subsequent relapse (72). Together, these findings suggest that habitual behavior is associated with alcohol dependence and may be relevant for treatment outcomes. Yet, we cannot draw conclusions regarding the effects of saracatinib on habitual behavior *per se* in the clinical study presented here. There is little work in the literature regarding back-translatability of effective treatments for alcohol use disorder in habit paradigms. One study assessed the effects of naltrexone on ethanol self-administration in rats using reinforcement schedules that promote goal-directed (FR-5) vs. habitual (VI-30) responding. They found that naltrexone reduced responding in both schedules, although they did not test effects of naltrexone on habit itself, such as in a contingency degradation or outcome devaluation session (73). More work is needed in this area to determine the translational potential of ethanol habit in rodents as a screen for novel therapeutics.

Overall, we did not identify effects of saracatinib on alcohol-seeking/craving or consumption in habitual mice or heavy drinking human participants. These results suggest that Fyn kinase inhibition may not be effective at reducing these aspects of alcohol use at the doses and treatment regimens employed in the current study. Future studies should consider the use of higher doses of saracatinib and alternative treatment regimens to confirm and expand upon these findings.

## Data Availability Statement

The raw data supporting the conclusions of this article will be made available by the authors, without undue reservation.

## Ethics Statement

The studies involving human participants were reviewed and approved by Yale Human Investigations Committee. The patients/participants provided their written informed consent to participate in this study. The animal study was reviewed and approved by Yale University Institutional Animal Care and Use Committee.

## Author Contributions

SK-S, JRT, JK, and SO'M: conceptualization. ST, CG, SO'M, DC, JS, JMT, KD, RG, BP, and SK-S: investigation and analyses. ST: writing original draft. ST, CG, SO'M, DC, JS, JMT, KD, RG, BP, JK, JRT, and SK-S: writing and editing. All authors contributed to the article and approved the submitted version.

## Author Disclaimer

The content is solely the responsibility of the authors and does not necessarily represent the official views of the National Institutes of Health, the Department of Mental Health and Addiction Services, or the state of Connecticut.

## Conflict of Interest

SO'M is a member of the American Society of Clinical Psychopharmacology's (ASCP's) Alcohol Clinical Trials Initiative, supported by Alkermes, Amygdala, Arbor Pharma, Dicerna, Ethypharm, Indivior, Lundbeck, Mitsubishi Tanabe, Otsuka; Consultant/advisory board member, Alkermes, Amygdala, Dicerna, Opiant; Medication supplies, Astra Zeneca, Novartis; DSMB member, Indiana University, Emmes Corporation. SK-S, PhD has received medication supplies from Astra Zeneca, Novartis. JK has been a consultant (< $10,000/year) for the following: Aptinyx, Inc., Atai Life Sciences, AstraZeneca Pharmaceuticals, Biogen, Idec, MA, Biomedisyn Corporation, Bionomics, Limited (Australia), Boehringer Ingelheim International, Cadent Therapeutics, Inc., Clexio Bioscience, Ltd., COMPASS Pathways, Limited, United Kingdom, Concert Pharmaceuticals, Inc., Epiodyne, Inc., EpiVario, Inc., Greenwich Biosciences, Inc., Heptares Therapeutics, Limited (UK), Janssen Research & Development, Jazz Pharmaceuticals, Inc., Otsuka America Pharmaceutical, Inc., Perception Neuroscience, Holdings, Inc., Spring Care, Inc., Sunovion Pharmaceuticals, Inc., Takeda Industries, and Taisho Pharmaceutical Co., Ltd. JK serves on the scientific advisory board for: Biohaven Pharmaceuticals, BioXcel Therapeutics, Inc. (Clinical Advisory Board), Cadent Therapeutics, Inc. (Clinical Advisory Board), Cerevel Therapeutics, LLC, EpiVario, Inc., Eisai, Inc., Jazz Pharmaceuticals, Inc., Lohocla Research Corporation, Novartis Pharmaceuticals Corporation, PsychoGenics, Inc., RBNC Therapeutics, Inc., Tempero Bio, Inc., Terran Biosciences, Inc. JK holds stock in: Biohaven Pharmaceuticals, Sage Pharmaceuticals, Spring Care, Inc. JK has stock options in: Biohaven Pharmaceuticals Medical Sciences, EpiVario, Inc., RBNC Therapeutics, Inc., Terran Biosciences, Inc., Tempero Bio, Inc. JK has received income (>$10,000) from their position as Editor - Biological Psychiatry. JK holds the following patents and inventions: 1. Seibyl JP, Krystal JH, Charney DS. Dopamine and noradrenergic reuptake inhibitors in treatment of schizophrenia. US Patent #:5,447,948.September 5, 1995. 2. Vladimir, Coric, Krystal, John H, Sanacora, Gerard – Glutamate Modulating Agents in the Treatment of Mental Disorders. US Patent No. 8,778,979 B2 Patent Issue Date: July 15, 2014. US Patent Application No. 15/695,164: Filing Date: 09/05/2017. 3. Charney D, Krystal JH, Manji H, Matthew S, Zarate C., - Intranasal Administration of Ketamine to Treat Depression United States Patent Number: 9592207, Issue date: 3/14/2017. Licensed to Janssen Research & Development. 4. Zarate, C, Charney, DS, Manji, HK, Mathew, Sanjay J, Krystal, JH, Yale University “Methods for Treating Suicidal Ideation,” Patent Application No. 15/379,013 filed on December 14, 2016 by Yale University Office of Cooperative Research. 5. Arias A, Petrakis I, Krystal JH. – Composition and methods to treat addiction. Provisional Use Patent Application no.61/973/961. April 2, 2014. Filed by Yale University Office of Cooperative Research. 6. Chekroud, A., Gueorguieva, R., & Krystal, JH. “Treatment Selection for Major Depressive Disorder” (filing date 3rd June 2016, USPTO docket number Y0087.70116US00). Provisional patent submission by Yale University. 7. Gihyun, Yoon, Petrakis I, Krystal JH – Compounds, Compositions and Methods for Treating or Preventing Depression and Other Diseases. U. S. Provisional Patent Application No. 62/444,552, filed on January10, 2017 by Yale University Office of Cooperative Research OCR 7088 US01. 8. Abdallah, C, Krystal, JH, Duman, R, Sanacora, G. Combination Therapy for Treating or Preventing Depression or Other Mood Diseases. U.S. Provisional Patent Application No. 62/719,935 filed on August 20, 2018 by Yale University Office of Cooperative Research OCR 7451 US01. 9. John Krystal, Godfrey Pearlson, Stephanie O'Malley, Marc Potenza, Fabrizio Gasparini, Baltazar Gomez-Mancilla, Vincent Malaterre. Mavoglurant in treating gambling and gaming disorders. U.S. Provisional Patent Application No. 63/125,181filed on December 14, 2020 by Yale University Office of Cooperative Research OCR 8065 US00. JK received the drug, Saracatinib from AstraZeneca Pharmaceuticals for research related to NIAAA grant “Center for Translational Neuroscience of Alcoholism” (CTNA-4). JK received the drug, Mavoglurant, from Novartis for research related to NIAAA grant “Center for Translational Neuroscience of Alcoholism” (CTNA-4). RG discloses the following interests unrelated to this work: royalties from book “Statistical Methods in Psychiatry and Related Fields” published by CRC Press, honorarium as a member of the Working Group for PTSD Adaptive Platform Trial of Cohen Veterans Bioscience and a United States patent application 20200143922 by Yale University: Chekroud, A., Krystal, J., Gueorguieva, R. and Chandra, A. “Methods and Apparatus for Predicting Depression Treatment Outcomes.” The remaining authors declare that the research was conducted in the absence of any commercial or financial relationships that could be construed as a potential conflict of interest.

## Publisher's Note

All claims expressed in this article are solely those of the authors and do not necessarily represent those of their affiliated organizations, or those of the publisher, the editors and the reviewers. Any product that may be evaluated in this article, or claim that may be made by its manufacturer, is not guaranteed or endorsed by the publisher.
