# Supplementary figures and images for: Saracatinib Fails to Reduce Alcohol-Seeking and Consumption in Mice and Human Participants
Source: Front Psychiatry. 2021 Aug 31;12:709559. doi: 10.3389/fpsyt.2021.709559 (PMC8438169; doi:10.3389/fpsyt.2021.709559)

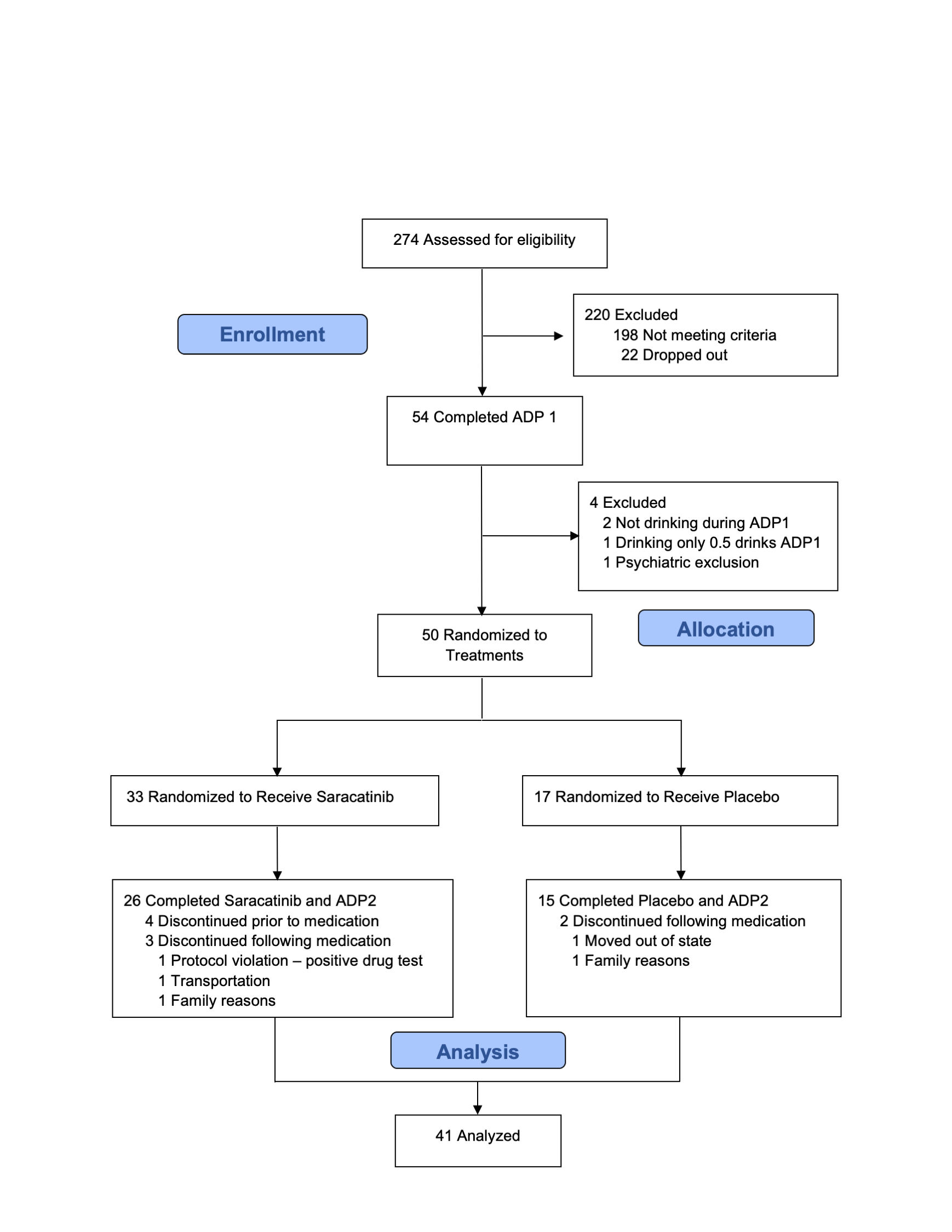

Supplement: Supplementary file 1 [file Image_1.TIFF]

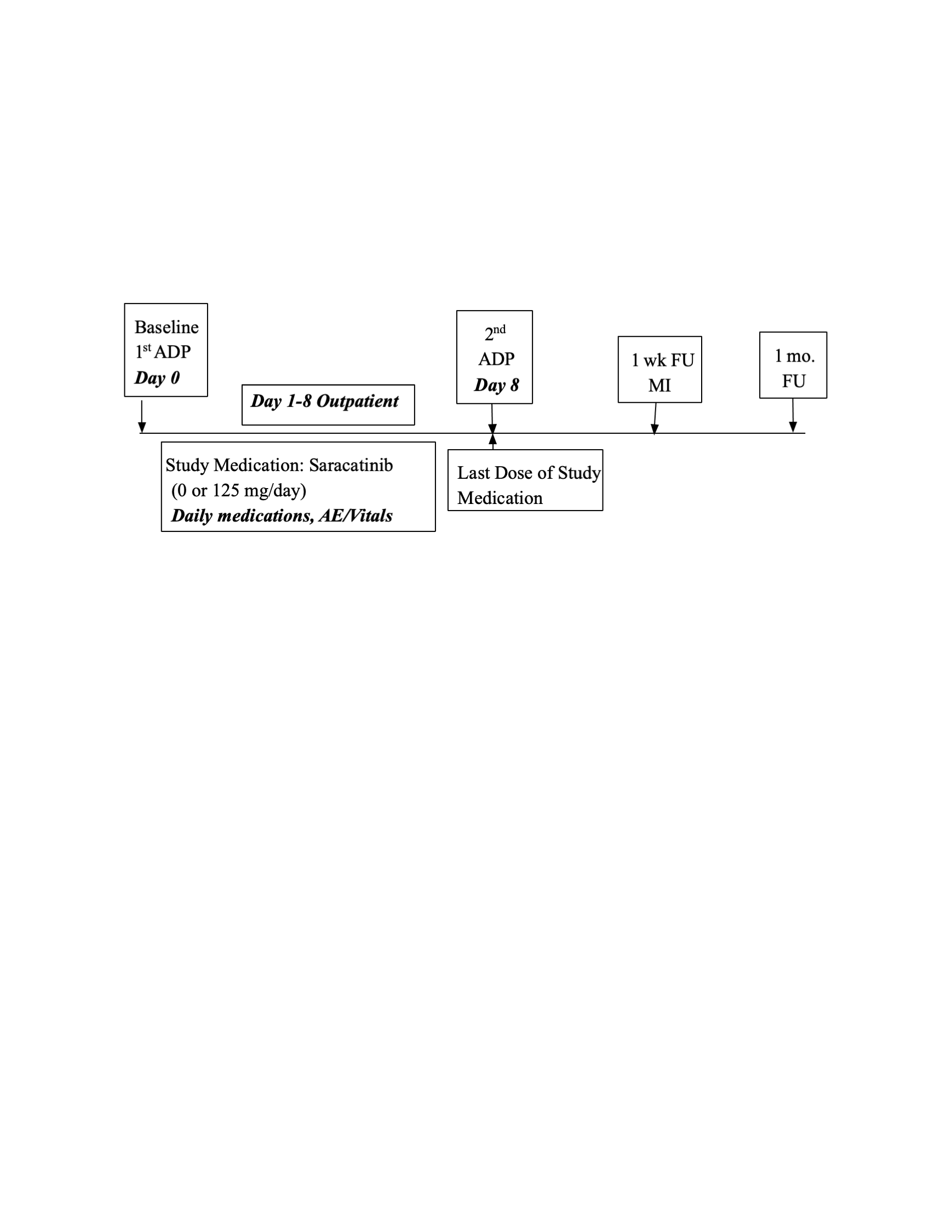

Supplement: Supplementary file 2 [file Image_2.TIFF]

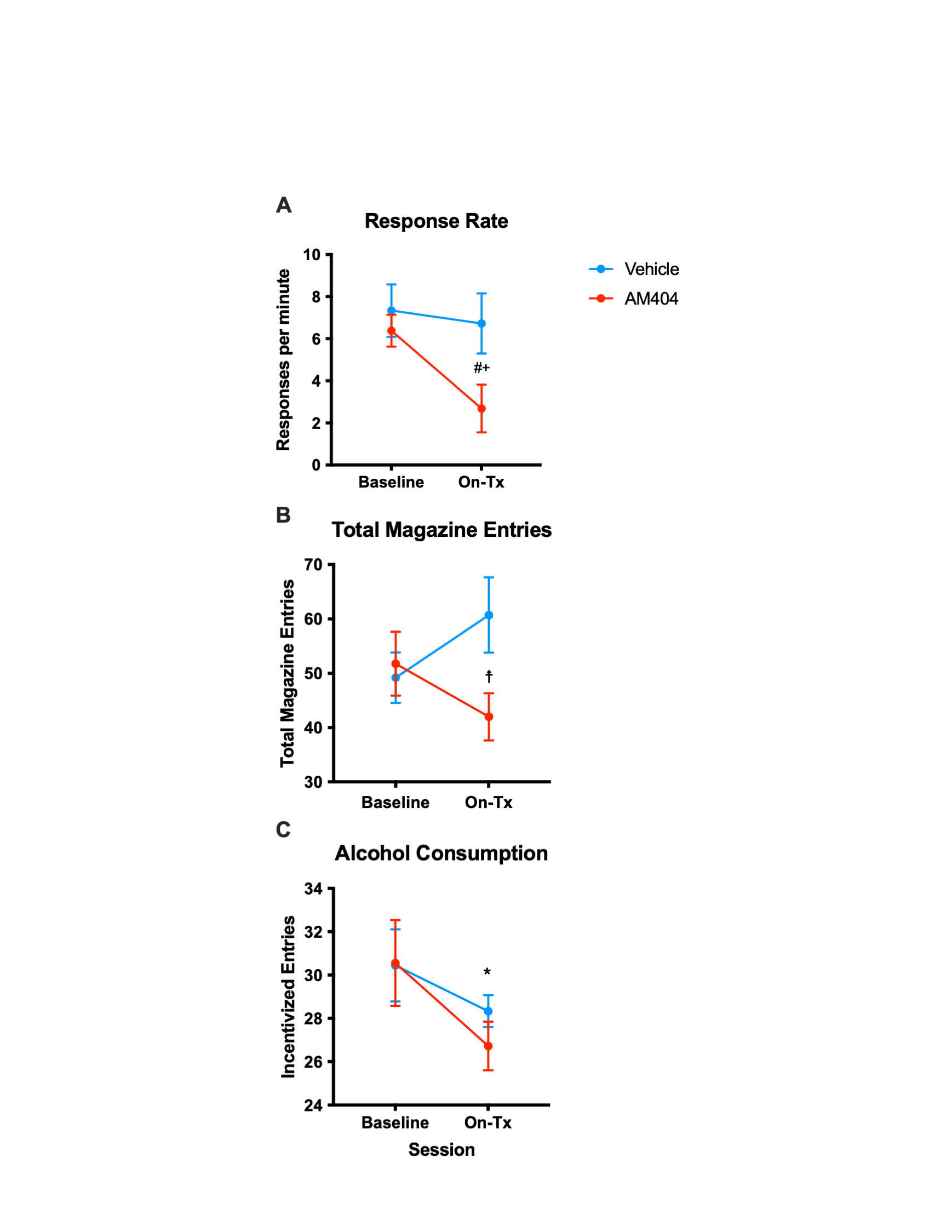

Supplement: Supplementary file 3 [file Image_3.TIFF]
