# Supplementary material for: Saracatinib Fails to Reduce Alcohol-Seeking and Consumption in Mice and Human Participants
Source: Front Psychiatry. 2021 Aug 31;12:709559. doi: 10.3389/fpsyt.2021.709559 (PMC8438169; doi:10.3389/fpsyt.2021.709559)
Supplement: Supplementary file 4 [file Data_Sheet_1.pdf]

Supplementary Material

RESULTS

Mouse Study

10 mg/kg AM404 reduced the number of active responses during the contingency degradation test (session×drug interaction: $\chi^2_{(1)} = 3.83$, $p = 0.05$; Fig. S3A) relative to the baseline session ($p<0.01$) and trended toward a reduction relative to the vehicle condition ($p = 0.07$). In contrast, vehicle administration did not affect responding during contingency degradation ($p = 0.94$). Similarly, AM404 reduced the number of magazine entries during contingency degradation (session×drug: $\chi^2_{(1)} = 5.57$, $p<0.05$; Fig. S3B) relative to the vehicle condition ($p = 0.001$). Incentivized magazine entries were reduced during contingency degradation, regardless of drug condition ($\chi^2_{(1)} = 4.77$, $p<0.05$; Fig. S3C). Mice earned an average of 1.14 ± 0.12 g/kg ethanol during AM404 testing.

Human Clinical Trial

Figure S2 illustrates the flow of participants through the study. Following initial eligibility determination, 54 participants completed the baseline ADP. Of these, three were excluded because they consumed less than one drink during the baseline ADP. One additional participant was excluded based on psychiatric criteria. Thus, the final sample was composed of 50 participants who were randomized 2:1 to receive either placebo (n = 17) or saracatinib (n = 33), respectively. Prior to the on-treatment ADP, 2 participants receiving placebo and 7 participants receiving saracatinib dropped out of the study, resulting in a total of 41 participants that completed all parts of the study (placebo: n = 15; saracatinib: n = 26).

CAPTIONS

Fig. S1: CONSORT diagram depicting the flow of participants through the study.

Fig. S2: Timeline for the human clinical trial. Participants completed the baseline ADP on Day 0, then underwent seven to eight days of 125 mg/day saracatinib administration in an outpatient setting. On Day 8, participants were administered the final dose of saracatinib and underwent the on-treatment ADP. Follow up occurred at one week and one month after the on-treatment ADP session. ADP: Alcohol Drinking Paradigm. AE: adverse events. FU: follow up. MI: motivational intervention.

Fig. S3: AM404 reduced habitual responding for ethanol. Mice underwent contingency degradation ("On-Tx" session) 30 minutes after administration of 10 mg/kg AM404 or vehicle in a within-subject, counterbalanced order. All mice received vehicle prior to the preceding VI-60 session ("Baseline"). (**A**) Response rate, (**B**) total magazine entries, and (**C**) incentivized magazine entries. N = 18 mice, within-subject. +p<.10 versus vehicle within session. #p<0.05 versus baseline session within drug condition. †p<0.05 versus vehicle within session. *p<0.05 versus baseline day across groups (main effect of session). Tx: treatment.

Table S1: Frequency of observed adverse events for the saracatinib group. Sum of mild, moderate, and severe may not add up to sum of number of participants reporting if a single participant experiences >1 severity rating for that event. MedDRA: Medical Dictionary for Regulatory Activities. SOC: system organ class. PT: preferred term.

Table S2. Frequency of observed adverse events for the placebo group. Sum of mild, moderate, and severe may not add up to sum of number of participants reporting if a single participant experiences >1 severity rating for that event. MedDRA: Medical Dictionary for Regulatory Activities. SOC: system organ class. PT: preferred term.

Table S1. Frequency of observed adverse events for saracatinib (n = 33)

MedDRA SOC<br>*e.g. Nervous system disorders*	MedDRA PT<br>*e.g. Headache*	Participants<br>n (%)	All severities<br>Mild<br>n (%)	 Moderate<br>n (%)	 Severe<br>n (%)
Gastrointestinal	Abdominal discomfort/pain	3 (9)	3 (9)	2 (6)	0 (0)
	Diarrhea	3 (9)	3 (9)	1 (3)	0 (0)
	Increased appetite	1 (3)	1 (3)	0 (0)	0 (0)
	Indigestion	1 (3)	1 (3)	0 (0)	0 (0)
	Nausea	5 (15)	4 (12)	1 (3)	0 (0)
	Vomiting	1 (3)	0 (0)	1 (3)	0 (0)
Central Nervous System/Psychiatric	Decreased sex drive	1 (3)	1(3)	0 (0)	0 (0)
	Dizziness	1 (3)	1(3)	0 (0)	0 (0)
	Fatigue	2 (6)	1(3)	1 (3)	0 (0)
	Feel high	0 (0)	0 (0)	0 (0)	0 (0)
	Headache	5 (15)	4 (12)	3 (9)	0 (0)
	Insomnia	0 (0)	0 (0)	0 (0)	0 (0)
	Somnolence	0 (0)	0 (0)	0 (0)	0 (0)
Ear, Nose, Throat	Cold symptoms	6 (18)	6 (18)	3 (9)	0 (0)
	Dehydrated	1 (3)	0 (0)	1 (3)	0 (0)
	Nasal congestion	4 (12)	4 (12)	2 (6)	0 (0)
	Sore throat	2 (6)	2 (6)	1 (3)	0 (0)
Musculoskeletal	Joint pain	3 (9)	2 (6)	1 (3)	0 (0)
Skin	Eczema	1 (3)	1 (3)	0 (0)	0 (0)
	Pruritus	1 (3)	1 (3)	0 (0)	0 (0)
Cardio/Pulmonary	Chest pain	1 (3)	1 (3)	0 (0)	0 (0)
	Tachycardia	2 (6)	1 (3)	1 (3)	0 (0)

Table S2. Frequency of observed adverse events for placebo (n = 17)

MedDRA SOC<br>*e.g. Nervous system disorders*	MedDRA PT<br>*e.g. Headache*	Participants<br>n (%)	All severities<br>Mild<br>n (%)	Moderate<br>n (%)	Severe<br>n (%)
Gastrointestinal	Abdominal discomfort/pain	1 (6)	1 (6)	0 (0)	0 (0)
	Diarrhea	2 (12)	1 (6)	1 (6)	0 (0)
	Increased appetite	0 (0)	0 (0)	0 (0)	0 (0)
	Indigestion	0 (0)	0 (0)	0 (0)	0 (0)
	Nausea	1 (6)	0 (0)	1 (6)	0 (0)
	Vomiting	2 (12)	1 (6)	1 (6)	0 (0)
Central Nervous System/Psychiatric	Decreased sex drive	0 (0)	0 (0)	0 (0)	0 (0)
	Dizziness	1 (6)	0 (0)	1 (6)	0 (0)
	Fatigue	3 (18)	3 (18)	1 (6)	0 (0)
	Feel high	1 (6)	1 (6)	0 (0)	0 (0)
	Headache	1 (6)	1 (6)	0 (0)	0 (0)
	Insomnia	1 (6)	1 (6)	0 (0)	0 (0)
	Somnolence	0 (0)	0 (0)	0 (0)	0 (0)
Ear, Nose, Throat	Cold symptoms	1 (6)	1 (6)	0 (0)	0 (0)
	Dehydrated	0 (0)	0 (0)	0 (0)	0 (0)
	Nasal congestion	0 (0)	0 (0)	0 (0)	0 (0)
	Sore throat	0 (0)	0 (0)	0 (0)	0 (0)
Musculoskeletal	Joint pain	0 (0)	0 (0)	0 (0)	0 (0)
Skin	Eczema	0 (0)	0 (0)	0 (0)	0 (0)
	Pruritus	0 (0)	0 (0)	0 (0)	0 (0)
Cardio/Pulmonary	Chest pain	0 (0)	0 (0)	0 (0)	0 (0)
	Tachycardia	2 (12)	1 (6)	1 (6)	0 (0)
